# Beyond Biological Sex: Interactive Effects of Gender Role and Sex Hormones on Spatial Abilities

**DOI:** 10.3389/fnins.2019.00675

**Published:** 2019-07-09

**Authors:** Belinda Pletzer, Julia Steinbeisser, Lara van Laak, TiAnni Harris

**Affiliations:** ^1^Department of Psychology, University of Salzburg, Salzburg, Austria; ^2^Centre for Cognitive Neuroscience, University of Salzburg, Salzburg, Austria

**Keywords:** gender role, sex hormones, mental rotation, navigation, sex differences

## Abstract

Sex differences in spatial abilities are well documented, even though their underlying causes are poorly understood. Some studies assume a biological basis of these differences and study the relationship of sex hormone levels to spatial abilities. Other studies assume social influences and study the relationship of gender role (masculinity/femininity) to spatial abilities. Contemporary theories postulate a psychobiosocial model of sex differences in spatial abilities, in which both biological (e.g., hormonal) and psychosocial (e.g., gender role) variables interactively modulate spatial abilities. However, few studies have addressed both aspects simultaneously. Accordingly, the present study explores potential interactive effects between gender role and sex hormones on spatial performance. 41 men and 41 women completed a mental rotation and a virtual navigation task. Sex hormone levels and gender role were assessed in all participants. Sex differences favoring men were observed in both tasks. We found that neither sex hormones nor gender role alone emerged as mediators of these sex differences. However, several interactive effects between gender role and sex hormones were identified. Combined effects of masculinity and testosterone were observed for those variables that displayed sex differences. Participants with both, high masculinity and high testosterone showed the best performance. However, this association was further modulated by biological sex and progesterone levels. Furthermore, we observed an interactive effect of femininity, estradiol and testosterone on response times in both tasks. Consistent across both tasks and irrespective of biological sex, testosterone related to response times in participants with low estradiol levels, depending on their femininity. In participants with low femininity, testosterone was related to slower reaction times, while in participants with higher femininity, testosterone was related to faster reaction times.

## Introduction

Sex differences have attracted considerable research interest over the past decade, but their underlying mechanisms remain yet to be uncovered. Some researchers see sex differences in adults as the direct result of organizational or activational effects of sex hormones, i.e., effects of sex hormones on the brain that occur either during fetal development or later in life (e.g., Kelly et al., 1999). Other researchers see sex differences in adults as a result of socialization and experience (e.g., Nash, 1979; Eagly and Koenig, 2006). With increasing age, children are more and more exposed to societal views of what’s acceptable or desirable for their biological sex (gender role/sex role). They develop their gender identity and a sense of how much they conform to these gender roles (e.g., Eagly and Koenig, 2006). The extent to which individuals conform to male gender roles is referred to as masculinity. The extent to which they conform to female gender roles is referred to as femininity.

Sex differences have been described for various domains including spatial, verbal and memory abilities (see Andreano and Cahill, 2009 for a review). While sex differences in some areas are more disputed than others, the predominant view is that some abilities are better developed in women, while other abilities are better developed in men (e.g., Halpern, 2000). The most robust sex differences have been described in the spatial domain, in which men on average outperform women (e.g., Levine et al., 2016). However, an important observation in sex difference research is their task specificity, since different – seemingly similar tasks – may involve a variety of different cognitive processes (Andreano and Cahill, 2009). Accordingly, the male superiority in spatial tasks is by no means universal. For instance, women outperform men in tasks of object location memory (see Voyer et al., 2007 for a meta-analysis). However, men appear to have a robust advantage in tasks of spatial visualization, like mental rotation tasks and navigation tasks (Andreano and Cahill, 2009). Sex differences in mental rotation and navigation have been described across different cultures (Silverman and Eals, 1992) and emerge with moderate to large effect sizes (0.5–1.3) in meta-analyses (e.g., Linn and Petersen, 1985; Voyer et al., 1995).

Accordingly, most research on the question of whether sex differences are of biological or societal origin, has focused on sex differences in these tasks (compare Levine et al., 2016). An important question in that regard concerns the development of sex differences in spatial visualization (Linn and Petersen, 1985; Levine et al., 2016). If sex differences in tasks of spatial rotation or navigation are already observed in early childhood, they may be the result of organizational effects of sex hormones. If they arise with the onset of puberty, they may be the result of activational effects of sex hormones. If they emerge at school age or after puberty, this may provide stronger support for societal influences. While children are first introduced to societal gender roles at home, the extent to which gender roles are enforced by parents varies greatly depending on their own views. With school age, however, gender role expectations are enforced by classmates and teachers, leading to a stronger and more homogenous exposure to societal gender roles at that age. However, a recent study places the onset of strongest enforcement of gender roles around the age of ten, linking it to an increased concern about girl’s sexuality and safety during puberty (Blum et al., 2017). Thus, endocrinological and social aspects of puberty are invariably confounded, making it hard to disentangle activational effects of sex hormones and societal influences during that time period.

Nevertheless, a variety of studies have focused on sex differences in spatial visualization in children and adolescents of various age groups. However, different studies arrive at different conclusions. While some studies do indeed report sex differences in mental rotation already in infants (Moore and Johnson, 2008, 2011; Quinn and Liben, 2008; Lauer et al., 2015) and preschoolers (Levine et al., 1999; Frick et al., 2013a), other studies are unable to find sex differences in spatial abilities in young children (Leplow et al., 2003; Frick et al., 2013b). However, studies in children require the use of age-appropriate tasks and the comparability of the tasks used in children to the tasks used in adults has been criticized (Andreano and Cahill, 2009; Levine et al., 2016). For instance, infant studies rely on different looking times between figures and their mirror image, and it is unclear, whether such looking patterns reflect spatial abilities. Furthermore, mental rotation tasks used in children are mostly two-dimensional, while adult MRT are three-dimensional. Most studies locate the emergence of sex differences in spatial abilities around puberty (Voyer et al., 1995; Andreano and Cahill, 2009; Titze et al., 2010). However, Linn and Petersen (1985) argue in their meta-analytic review, that the emergence of sex differences in spatial abilities is likely later around the age of 18, which they link to a stronger age-related increase in spatial abilities in boys compared to girls. However, since this point is far past the onset of puberty, the increase in boy’s spatial abilities cannot be explained by activational effects of sex hormones. It is, however, possible, that young adults experience an increased exposure to gender role expectations with the transition to independence at the age of 18. This is usually the time during which they choose a career and – in many countries – boys undergo military training. These factors may not only contribute to enforce the societal views of what’s male or female, but also lead to increased training of young men in spatial abilities. Numerous studies indicate that spatial skills are highly trainable and that training reduces sex differences in spatial tasks (see Levine et al., 2016 for a review).

Furthermore, findings of hormone-related changes in cognitive functions, including spatial functions, along the female menstrual cycle (e.g., Hausmann et al., 2000), during pregnancy (Workman et al., 2012) or menopause (e.g., Halbreich et al., 1995), suggest that sex hormones continuously reshape our brain throughout our adult life. Accordingly, pinpointing the onset of sex differences in cognitive abilities to a certain age, may not be sufficient. If sex differences in adults are the result of different hormonal milieus between men and women, the actual level of circulating sex hormones at the time of testing should modulate sex differences in spatial abilities. However, studies relating circulating testosterone levels to spatial abilities in adults arrive at mixed results. While some studies observe linear or u-shaped relationships in men or women (e.g., Hooven et al., 2004; Driscoll et al., 2005; Hausmann et al., 2009; Courvoisier et al., 2013), other studies find no relationship of circulating testosterone levels to mental rotation performance (Halari et al., 2005; Falter et al., 2006; Puts et al., 2010). It is possible that these conflicting results arise from complex interactions between organizational and activational effects of sex hormones, i.e., activational effects of hormones may differ in differently organized neural structures. For instance it was recently observed that testosterone relates to hippocampal volumes in women, but not in men (Pletzer, 2019) and findings of testosterone showing different relationships to spatial abilities in men and women are not uncommon (Hooven et al., 2004; Driscoll et al., 2005; Hausmann et al., 2009; Courvoisier et al., 2013). However, not only biological sex, but also interactive effects between different sex hormones may play a role. Testosterone is converted to estradiol via the enzyme aromatase and into the physiologically more active dihydro-testosterone via the enzyme 5α-reductase. This enzyme does, however, show a higher affinity to progesterone, such that in the presence of high progesterone levels, less testosterone gets converted into dihydro-testosterone. Accordingly, testosterone effects may be alleviated in the presence of high progesterone.

Furthermore, if sex differences in adults are indeed the result of socialization by which individuals learn to adapt their behavior according to the societal views of what’s typical for a certain gender, the extent to which individuals have incorporated these roles into their self-image, should explain sex differences in spatial abilities. Indeed masculinity was found to relate positively to mental rotation performance in a recent meta-analysis (Reilly and Neumann, 2013), while femininity showed no such association. However, the majority of studies included in this meta-analyses have used the Bem sex role inventory (BSRI; Bem, 1974) to assess gender role. This measure has however been criticized due to its poor factorial validity on the one hand, the exclusion of relevant dimensions of gender role, such as activities and interests, and the fact that the item pool as collected in the 1970s appears outdated with respect to a more modern understanding of gender roles (e.g., Choi and Fuqua, 2003).

Contemporary theories assume that sex differences develop according to a psychobiosocial model, i.e., as a result of interactions between biological, e.g., hormonal, influences, societal influences and individual characteristics (e.g., Levine et al., 2016). Individuals differ in the extent to which they conform to societal expectations not only because of the way they are brought up, but also because of their personality. Furthermore, personality factors have also been known to affect how susceptible subjects are to fluctuations in their hormonal milieu (e.g., Gingnell et al., 2010; Stenbæk et al., 2019). Particularly higher scores of neuroticism have been related to a higher susceptibility to hormonal fluctuations in women, albeit this has mostly been studied with respect to mood changes. It is unclear whether the personality trait neuroticism reflects a certain brain organization that responds more strongly to hormonal changes or whether vice versa concurrent mood changes in response to hormonal fluctuations are perceived as more neurotic by participants. Nevertheless, these findings suggest that like activational effects of sex hormones, social influences act differently on differentially organized neural structures. In line with this assumption, it was recently observed that femininity relates to frontal gray matter volumes in men, but not in women (Pletzer, 2019). Particularly since conceptually there is some overlap between scales assessing neuroticism and scales assessing femininity, it is also plausible that social influences (e.g., gender role) amplify or diminish hormonal influences (both organizational and activational) on behavior.

However, only a few studies have addressed both biological and psychosocial factors in the same study. To the best of our knowledge, only one study has done so with respect to spatial abilities (Hausmann et al., 2009). They found interactive effects of sex hormones and stereotypes on spatial performance in the sense that testosterone mediated the effects of gender stereotypes in spatial abilities. In the present study, we address whether sex differences in mental rotation and spatial navigation are mediated via masculinity, femininity or sex hormones. We hypothesize that particularly testosterone levels and masculinity relate positively to spatial abilities and act as mediators for sex differences therein. In an integrative approach we additionally seek to identify interactive effects of biological sex, gender role, and sex hormones on spatial abilities. More specifically, we expect the best spatial abilities in participants with both, high masculinity and high testosterone levels, i.e., we expect masculinity to facilitate testosterone actions on spatial abilities. Furthermore, we expect stronger testosterone effects in participants with low progesterone levels.

## Materials and Methods

### Participants and Procedure

A total of 41 healthy young men and 45 healthy young women was recruited for the present study. All participants were between 18 and 35 years of age, had passed general qualification for university entrance and had no psychiatric, neurological or endocrinological disorders. Women did not take hormonal contraceptives, had no current or prior diagnosis of premenstrual dysphoric disorder and had a regular menstrual cycle of 21–35 days with no more than 7 days of variation between individual cycles (Fehring et al., 2006). Cycle-length and cycle regularity was established by participants self-reports of their last three onsets of menses. Test sessions for women were scheduled in the mid-luteal cycle phase, since some previous studies suggest that women behave most “female-like” during this cycle phase and the largest sex differences in spatial abilities have been reported for this phase (e.g., Hampson, 1990; Hausmann et al., 2000). The mid-luteal cycle phase spanned from 3 days post-ovulation up to 3 days before the expected onset of participants next menses. Ovulation was calculated by subtracting 14 days from the expected onset of next menses according to the participant’s last onset of menses and cycle length as based on the past three cycles. Onset of next menses was evaluated in follow-up.

Upon arrival at the lab, participants were asked to rinse their mouth, signed the informed written consent for the study and completed a general health related screening questionnaire. They then gave the first saliva sample. Afterward, they completed the computerized mental rotation task (MRT). After the MRT, participants gave their second saliva sample. Then they completed the virtual navigation task (VNT). Upon completion of the navigation task, participants gave a third saliva sample and completed questionnaires regarding the video-gaming experience, the gender related attributes questionnaire, the masculinity and femininity self-report scales, as well as the screening version of Ravens Advanced Progressive Matrices (APM; Raven et al., 1962) to obtain an estimate of IQ. Gender role scales were scheduled after the spatial tasks and after the last saliva samples in order to avoid any stereotype threat like influences by briefing participants about gender role. After debriefing the participants they received either course credits or monetary enumeration.

### Ethics Statement

The study was approved by the University of Salzburg’s ethics committee and conforms to the Code of Ethics of the World Medical Association (Declaration of Helsinki). Informed written consent was obtained from all participants.

### Assessment of Spatial Abilities

#### Mental Rotation Task (MRT)

For the mental rotation task, 30 items were selected from the Ganis and Kievit (2015) stimulus library. Participants were presented with two three-dimensional figures. Their task was to decide whether the two figures were the same, but rotated, or whether the two figures were different, as fast and accurately as possible within a pre-specified time-limit of 7 s. 15 items required a *“same”* response (left mouse button), 15 items a *“different”* response (right mouse button). Same figures were rotated by 50° (5 items), 100° (5 items) or 150° degrees (5 items). Different figures were mirror images of the same figures and rotated by the same degree. The order of stimuli was randomized in each participant. Reaction time (RT) and accuracy were recorded for each item.

#### Virtual Navigation Task (VNT)

The navigation task used in the present study was a virtual reality (VR) adaptation of the task used in Harris et al. (2019). Ten items were selected from the task developed by Harris et al. (2019) using Unreal Engine 4 18.3 and presented to participants via a HTC Vive virtual reality system. Each item represented a new environment consisting of a 10 × 10 matrix with different landmarks placed on each field. Participants were given three lines of directions to a target location in the environment and their task was to reach the target location as quickly as possible. There was no pre-specified time-limit to complete each item. Participants could only move on to the next item, once they found the target location. All directions used allocentric terms (“north,” “south,” “east,” and “west”) to guide participants through the environment and participants learned which direction they were facing at the beginning of each item. Furthermore, half of the items used landmark-terms to guide participants (e.g., “go to the tree”), the other half used Euclidian terms (e.g., “go for four blocks”). For each item, the time participants needed to reach the target location (navigation time) was recorded.

### Assessment of Gender Role

Two measures were used to assess gender role: (i) self-ratings of masculinity and femininity. (ii) the gender-related attributes questionnaire as an objective measure of personality traits, cognitive abilities and interests that are (stereo-)typically associated with men or women.

#### Gender Role Self-Assessment

On a nine-point Likert-Scale, participants were asked to rate how masculine or feminine they perceived themselves. Since men tend to compare themselves to other men, while women tend to compare themselves to other women (Pletzer et al., 2015), each rating was performed three times: (i) in comparison to (other) men, (ii) in comparison to (other) women, and (iii) in comparison to the general population. The same scale was already employed by Pletzer et al. (2015). These ratings represent subjective measures of masculinity and femininity and depend on the participant’s personal understanding of these concepts. As outlined by Pletzer et al. (2015) the concepts of masculinity and femininity vary between cultures and possibly also subcultures, e.g., depending on education or generation.

#### Gender-Related Attributes Scale (GERAS)

As a more objective measure of masculinity and femininity, we recently developed the gender related attributes scale (GERAS; Gruber et al., 2019). The GERAS assesses gender role via attributes that are typically perceived as masculine or feminine in middle European cultures. It extends previous sex role inventories (e.g., BSRI; Bem, 1974) by including not only personality traits, but also cognitive abilities and interests typically associated with the male or female gender, and thus spans multiple aspects of gender roles. Accordingly it consists of three subscales: (i) personalities subscale with 20 items (10 masculine and 10 feminine), (ii) cognitions subscale with 14 items (7 masculine and 7 feminine), and (iii) interests subscale with 16 items (8 masculine and 8 feminine). In the personality subscale participants are asked to rate how often in their opinion positive and negative traits typically associated with the male (e.g., dominant and bold) or female (e.g., warm-hearted and sensitive) gender apply to them. In the cognitions subscale participants are asked to rate how well they think they are able to perform certain tasks, for which previous studies have demonstrated sex differences favoring men (e.g., find a way) or women (e.g., find the right words). In the interests subscale participants are asked to rate how interested they would be to engage in activities which are stereotypically preferred by men (e.g., boxing and drinking) or women (e.g., dancing and talking). All ratings are performed on a seven-point Likert-scale. Separate masculinity and femininity scores can be obtained for each subscale by averaging the ratings for masculine and feminine items, respectively. Overall masculinity and femininity scores are obtained by averaging the masculinity and femininity scores of the three subscales. The GERAS has been well-validated and shows excellent internal consistency and reliability (Gruber et al., 2019). In particular, masculinity and femininity scores obtained with the GERAS are highly correlated to participant’s masculinity and femininity self-assessment (Gruber et al., 2019).

Accordingly, composite measures of masculinity and femininity were obtained by averaging GERAQ and self-assessment scores after recoding self-assessment scores to a seven-point scale by collapsing the two extreme categories at both ends of the scale. The composite score does not only reflect how much participants identify with pre-dominant societal views of what’s male and what’s female, but also take into account their self-perceived masculinity and femininity based on their own views of what’s male and what’s female. To address, how much participants conform to the typical male vs. typical female dichotomy, a masculinity-to-femininity ratio was also calculated. The masculinity-to-femininity ratio was the higher the more typically male and the lower, the more typically female participants were.

### Assessment of Sex Hormones

As outlined in the procedures, three saliva samples were obtained throughout the study – one in the beginning of the experiment, one after the MRT and one after the VNT. All saliva samples were acquired before masculinity and femininity were assessed in order to avoid any effects of priming participants about gender role on sex hormones. Saliva samples were immediately frozen at −20° after the experiment and centrifuged twice for 15 and 10 min at 3000 rpm, respectively. As recommended by the ELISA kit instructions, the three samples of each participant were pooled to account for fluctuations in hormone and saliva production. Estradiol, progesterone and testosterone were assessed from the pooled samples using DeMediTec salivary ELISA kits. While the approach to pool samples certainly has the advantage of providing a more stable measure of the average hormone concentrations throughout the experiment, hormonal variations in response to experimental manipulations are not taken into account. While effects of priming participants about gender roles were avoided by the order of measures (see procedure), it cannot be completely ruled out that the spatial tasks themselves elicited a hormonal response.

To reflect the activity of the enzyme aromatase, which converts testosterone to estradiol, an estradiol-to-testosterone ratio was calculated. Furthermore, testosterone is more physiologically active in its dehydrogenized form (dihydro-tetosterone) by conversion via the enzyme 5α-reductase. Since progesterone has a higher affinity to that enzyme than testosterone, testosterone is less physiologically active in the presence of high progesterone levels (e.g., Sitruk-Ware, 2006). Accordingly, to assess testosterone’s access to the enzyme 5α-reductase and obtain a measure of its physiological activity, a testosterone-to-progesterone ratio was calculated. Similarly, in women, estradiol actions are often counteracted by progesterone – possibly due to their opposite effects on a variety of neurotransmitter systems (Barth et al., 2015). Accordingly, to obtain a measure of free estrogenic activity, an estradiol-to-progesterone ratio was calculated.

### Statistical Analyses

**FIGURE 1:**
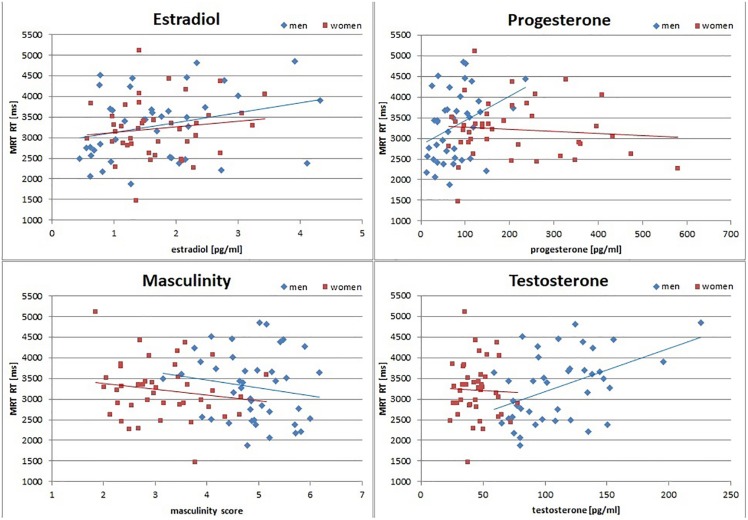
Relationship of sex hormones and masculinity to mental rotation reaction times (MRT RT). Estradiol was positively related to MRT RT in men and women, while testosterone and progesterone were positively related to MRT RT only in men. Note, however, that the association to testosterone survived across participants. Masculinity was negatively related to MRT RT in both men and women. Accordingly, in the MRT participants with higher levels of sex hormones responded slower, and participants with higher masculinity responded faster.

Statistical analyses were performed in SPSS 22 and R 3.5.1. As a manipulation check gender role, sex hormones, and spatial ability scores were compared between men and women using independent samples *t*-tests. To identify potential candidates for mediation analyses, interrelations between gender role, sex hormones and spatial abilities were assessed using Pearson correlations. In addition, partial correlations controlling for biological sex were performed to assess which variables related to spatial abilities, irrespective of biological sex. To assess, whether gender role or sex hormones were able to explain the sex difference in spatial abilities, mediation analyses were performed using the *mediate* function of the *mediation* packages (Tingley et al., 2017). To assess, whether biological sex, gender role and sex hormones interactively modulated spatial abilities, multiple regression analyses were performed. Details are described in the results section. To illustrate the combined or interactive effects of multiple variables in 3d space (Figures 1, 3, 4), we used the *gridfit* function in matlab 2016.

## Results

Four women had to be excluded because the onset of their next menses after the study was too early or too late and their progesterone values were below the acceptable range for the luteal cycle phase (<43 pg/ml; compare Harris et al., 2019). Hormone levels of one male participant were excluded from analysis as outliers, since they exceeded the group mean by more than three standard deviations. Accordingly data of 40 men and 41 women were used for analysis. Women’s average cycle length was 29 days (*SD* = 3 days). They were on average tested on day 22 of their cycle (*SD* = 4 days). An additional 5 participants (1 men and 4 women) did not complete the VNT task due to severe motion sickness.

### Sex Differences

Table 1 summarizes the descriptive statistics and gender comparisons for demographic data, sex hormones, gender role and spatial abilities. Men and women did not differ in age and IQ. Furthermore, there were no differences in estradiol levels between men and women (compare Table 1). As expected, men showed higher testosterone levels and masculinity ratings than women, while women showed higher progesterone levels and femininity ratings than men. Regarding spatial abilities, sex differences were observed for MRT accuracy and VNT RT, but not for MRT RT.

**TABLE 1:** Descriptives and biological sex differences for demographic variables, gender role, sex hormones, and spatial abilities.

	**men**		**Women**		**comparison**			
	**mean**	**SD**	**mean**	**SD**	**MD**	**d**	**t**	**p**
Age (years)	23.20	3.53	24.32	3.42	–1.12	–0.32	–1.46	0.15
IQ	112.54	7.95	109.29	8.59	3.24	0.39	1.78	0.08
Masculinity self (1–9)	6.37	1.15	3.20	1.56	3.17	2.31	10.45	< 0.001
Femininity self (1–9)	3.45	1.08	6.82	1.33	–3.37	–2.79	–12.62	< 0.001
Masculinity GERAQ [1–7]	4.54	0.90	3.90	0.77	0.64	0.77	3.48	< 0.001
Femininity GERAQ [1–7]	4.36	0.69	4.78	0.70	–0.42	–0.61	–2.77	0.007
Masculinity total (Z)	0.56	0.66	–0.56	0.62	1.12	1.78	8.04	< 0.001
Femininity total (Z)	–0.54	0.52	0.54	0.72	–1.11	–1.78	–8.07	< 0.001
Masc./fem. ratio (Z)	0.77	0.74	–0.77	0.53	1.54	2.40	10.87	< 0.001
Estradiol (E) (pg/ml)	1.71	0.98	1.72	0.69	–0.02	–0.02	–0.09	0.93
Progesterone (P) (pg/ml)	85.70	74.68	208.22	127.47	–122.53	–1.17	–5.31	< 0.001
Testosterone (T) (pg/ml)	114.94	43.93	44.82	13.19	70.12	2.16	9.79	< 0.001
E/T ratio (Z)	–0.71	0.40	0.69	0.93	–1.40	–1.94	–8.74	< 0.001
E/P ratio (Z)	0.63	1.03	–0.61	0.44	1.24	1.58	7.11	< 0.001
T/P ratio (Z)	0.72	1.00	–0.70	0.12	1.41	1.99	8.97	< 0.001
MRT Accuracy (%)	90.40	8.80	79.20	16.00	0.11	0.88	3.96	< 0.001
MRT RT (ms)	3286.85	815.67	3220.20	685.84	66.65	0.09	0.40	0.69
VNT RT (s)	76.80	50.52	115.60	72.62	–38.80	–0.63	–2.74	0.008

*GERAQ, gender-related attributes questionnaire; MRT, mental rotation task; VNT, virtual navigation task; RT, reaction time; MD, mean difference; d, Cohen’s d.*

### Relationship Between Gender Role, Sex Hormones, and Spatial Abilities

To identify potential mediators of sex differences in spatial abilities, Pearson correlations between gender role, sex hormones and their respective ratios and spatial abilities were calculated. Table 2 summarizes zero-order correlations between gender role, sex hormones and spatial abilities below the diagonal. Masculinity and testosterone levels were highly positively interrelated and both related to mental rotation accuracy and navigation time, but not mental rotation RT. Progesterone was positively related to femininity and both related negatively to MRT accuracy, but not RT or navigation time. Estradiol was not related to gender role, MRT accuracy or navigation time, but was positively related to MRT RT.

**TABLE 2:** Pearson and partial correlations between gender role, sex hormones, and spatial abilities.

	**Gender role**				**Sex hormones**					**MRT Acc.**	**MRT RT**	**VNT RT**
	**Masc.**	**Fem.**	**M/F**	**T**	**E**	**P**	**E/T**	**E/P**	**T/P**			
Masculinity (Masc)		–0.60^∗∗∗^	0.85^∗∗∗^	–0.13	–0.02	–0.06	0.03	0.08	0.03	0.10	–0.16	0.09
Femininity (Fem)	–0.83^∗∗∗^		–0.81^∗∗∗^	0.05	–0.04	0.05	–0.07	–0.02	0.02	0.04	–0.05	–0.10
Masc/Fem (M/F)	0.94^∗∗∗^	–0.92^∗∗∗^		–0.14	–0.04	< 0.01	0.04	0.02	0.03	–0.01	–0.10	0.13
Testosterone (T)	0.54^∗∗∗^	–0.59^∗∗∗^	0.55^∗∗∗^		0.55^∗∗∗^	0.31^∗∗^	–0.12	–0.08	–0.18	0.02	0.32^*^	–0.07
Estradiol (E)	–0.03	–0.01	–0.04	0.33^∗∗^		0.43^∗∗∗^	0.59^∗∗∗^	0.25^*^	–0.38^∗∗∗^	–0.15	0.23^*^	–0.07
Progesterone (P)	–0.46^∗∗∗^	0.47^∗∗∗^	–0.44^∗∗^	−0.28^*^	0.37^∗∗∗^		0.19	–0.39^∗∗∗^	–0.34^∗∗^	–0.17	0.04	–0.20
E/T	–0.51^∗∗∗^	0.52^∗∗∗^	–0.52^∗∗∗^	–0.60^∗∗∗^	0.44^∗∗∗^	0.51^∗∗∗^		0.23^*^	−0.24^*^	–0.17	0.06	0.06
E/P	0.52^∗∗∗^	–0.50^∗∗∗^	0.49^∗∗∗^	0.45^∗∗∗^	0.18	–0.61^∗∗∗^	–0.31^∗∗^		0.57^∗∗∗^	< 0.01	–0.09	–0.06
T/P	0.55^∗∗∗^	–0.55^∗∗∗^	0.56^∗∗∗^	0.47^∗∗∗^	−0.28^*^	–0.60^∗∗∗^	–0.62^∗∗∗^	0.76^∗∗∗^		0.04	–0.18	–0.05
MRT Accuracy	0.36^∗∗∗^	–0.29^∗∗^	0.31^∗∗^	0.33^∗∗^	–0.15	–0.36^∗∗∗^	–0.40^∗∗∗^	0.26^*^	0.32^∗∗^		–0.04	–0.09
MRT RT	–0.07	< 0.01	–0.02	0.24^*^	0.23^*^	< 0.01	0.01	–0.04	–0.09	–0.02		0.30^*^
VNT RT	–0.19	0.20	–0.17	−0.27^*^	–0.08	< 0.01	0.25^*^	−0.23^*^	−0.24^*^	0.21	0.27^*^	

Pearson correlations are displayed below the diagonal in light gray. Partial correlations after controlling for biological sex are displayed above the diagonal in white. MRT, mental rotation task; VNT, virtual navigation task; RT, reaction time. ^*^p < 0.05, ^∗∗^*p* < 0.01, and ^∗∗∗^*p* < 0.001.

To assess the interrelation between gender role and sex hormones and their relationship to spatial abilities irrespective of biological sex, partial correlations controlling for biological sex were performed, since gender role and sex hormones are inadvertently confounded with biological sex. Partial correlations are summarized above the diagonal in Table 2. After controlling for biological sex, the masculinity and femininity total score were significantly negatively interrelated, while sex hormones were significantly positively interrelated. There was no significant association between gender role and sex hormones. Gender role and sex hormones were not related to MRT accuracy or navigation time. Testosterone and estradiol both related positively to MRT RT. MRT RT and navigation time were significantly positively interrelated. Separate analyses by biological sex confirmed that these correlations were observed in both men and women, although the associations of sex hormones to MRT RT were significant only in men (Figure 1 and Table 3). Furthermore a positive correlation between progesterone and MRT RT was observed only in men (Figure 1).

**TABLE 3:** Pearson correlations between gender role, sex hormones, and spatial abilities for men and women.

	**Gender role**				**Sex hormones**					**MRT Acc.**	**MRT RT**	**VNT RT**
	**Masc.**	**Fem.**	**M/F**	**T**	**E**	**P**	**E/T**	**E/P**	**T/P**			
Masculinity (Masc)		–0.62^∗∗∗^	0.90^∗∗∗^	0.17	0.10	–0.09	–0.01	0.23	0.14	0.16	–0.16	–0.04
Femininity (Fem)	–0.59^∗∗^		–0.85^∗∗∗^	0.03	–0.14	0.11	–0.09	–0.30	–0.23	–0.02	0.12	–0.01
Masc/Fem (M/F)	0.85^∗∗∗^	–0.90^∗∗∗^		0.10	0.15	–0.07	0.05	0.27	0.17	0.14	–0.16	–0.05
Testosterone (T)	–0.29	0.09	–0.21		0.38^*^	0.48^∗∗∗^	−0.37^*^	–0.26	–0.01	< 0.01	–0.03	–0.24
Estradiol (E)	–0.12	0.06	–0.13	0.63^∗∗∗^		0.53^∗∗∗^	0.66^∗∗∗^	0.19	−0.37^*^	–0.24	0.13	–0.02
Progesterone (P)	–0.01	–0.20	0.13	0.48^∗∗∗^	0.51^∗∗^		0.16	–0.63^∗∗∗^	–0.74	–0.20	–0.10	–0.28
E/T	0.13	–0.01	0.04	–0.01	0.74^∗∗∗^	0.37^*^		0.38^*^	−0.38^*^	–0.18	0.06	0.10
E/P	0.03	0.17	–0.06	–0.05	0.27	–0.49^∗∗∗^	0.25		0.63^∗∗∗^	–0.07	0.07	0.23
T/P	0.03	0.08	0.02	–0.19	–0.44	–0.72^∗∗∗^	–0.53^∗∗∗^	0.59^∗∗∗^		0.10	–0.07	0.03
MRT Accuracy	–0.04	0.23	–0.19	0.03	–0.07	–0.03	–0.13	0.06	0.07		0.10	–0.09
MRT RT	–0.16	–0.03	0.05	0.46^∗∗^	0.29	0.35^*^	0.07	–0.15	–0.23	–0.26		0.31
VNT RT	0.27	–0.26	0.30	< 0.01	–0.13	0.04	–0.05	–0.25	–0.10	–0.11	0.32^*^	

Pearson correlations for men are displayed below the diagonal in light gray. Pearson correlations for men are displayed above the diagonal in white. MRT, mental rotation task; VNT, virtual navigation task; RT, reaction time. ^*^p < 0.05, ^∗∗^*p* < 0.01, and ^∗∗∗^*p* < 0.001.

### Mediation Analyses

To address, whether gender role or any of the sex hormones mediated the sex differences in spatial performance, mediation analyses were performed. In all except two analyses, the direct effect of sex remained significant, while the indirect effect never reached significance (Table 4). Exceptions were masculinity and testosterone in the navigation task. Controlling for masculinity or testosterone the direct effect of sex was not significant anymore. However, the indirect effect also did not reach significance. Accordingly, neither gender roles nor sex hormones mediated the sex difference in MRT accuracy and navigation time.

**TABLE 4:** Direct and mediated effects of sex on spatial abilities.

		**age**	**IQ**	**masc**	**Fem**	**M/F**	**T**	**E**	**P**	**E/T**	**E/P**	**T/P**
MRT Accuracy	Indirect	–0.03	–0.05	–0.20	0.09	0.01	0.04	–0.01	–0.22	–0.29	< 0.01	–0.08
	Direct	–0.77^∗∗∗^	–0.76^∗∗^	−0.59^*^	–0.90^∗∗∗^	−0.82^*^	–0.78^∗∗^	–0.82^∗∗∗^	−0.60^*^	−0.53^∼^	–0.82^∗∗∗^	–0.74^∗∗^
	Total	–0.81^∗∗∗^	–0.81^∗∗∗^	–0.79^∗∗∗^	–0.81^∗∗∗^	–0.81^∗∗∗^	–0.81^∗∗∗^	–0.82^∗∗∗^	–0.82^∗∗∗^	–0.83^∗∗∗^	–0.82^∗∗∗^	–0.82^∗∗∗^
	%mediated	0.03	0.05	0.25	–0.10	–0.02	–0.04	< 0.01	0.26	0.36	< 0.01	0.09
VNT RT	Indirect	< 0.01	0.07	–0.22	–0.28	–0.33	0.15	0.006	–0.25	0.11	0.10	0.11
	Direct	0.60^∗∗^	0.54^*^	0.82^*^	0.88^*^	0.92^*^	0.45	0.59^*^	0.85^∗∗^	0.48	0.49	0.47
	Total	0.60^∗∗^	0.60^∗∗^	0.60^∗∗^	0.60^∗∗^	0.59^∗∗^	0.60^∗∗∗^	0.59^*^	0.61^∗∗^	0.59^∗∗^	0.59^∗∗^	0.58^*^
	%mediated	< 0.01	0.10	–0.34	–0.44	–0.56	0.25	0.003	–0.40	0.18	0.16	0.18

Sex differences were not mediated by demographic variables, gender role or sex hormones. MRT, mental rotation task; VNT, virtual navigation task; RT, reaction time; Masc, masculinity; fem, femininity; T, testosterone; E, estradiol; P, progesterone. ^∼^*p* < 0.10, ^*^p < 0.05, ^∗∗^*p* < 0.01, and ^∗∗∗^*p* < 0.001.

### Beyond Biological Sex Differences: Interactive Effects of Gender Role and Sex Hormones

To explore interactive effects of gender role and sex hormones on spatial abilities, we used a data driven approach. For masculinity and femininity, respectively, models including all interactions with biological sex and sex hormones were defined and non-significant interactions were backward eliminated from higher to lower orders, such that only significant interactions and their lower order terms remained in the model beyond the main effects. Table 5 summarizes results for masculinity, while Table 6 summarizes the results for femininity.

**TABLE 5:** Results of exploratory multiple regression models including interactions between biological sex, masculinity, and sex hormones.

	**MRT accuracy**		**MRT RT**		**VNT RT**	
	**b**	**t**	**b**	**t**	**b**	**t**
Masculinity (Masc)	0.87	2.57^*^	–0.28	−2.18^*^	–0.03	–0.14
Testosterone (T)	0.63	1.72^∼^	0.39	3.06^∗∗^	–0.44	–1.74
Masc^*^T	1.18	3.00^∗∗^			0.12	0.69
Sex	0.10	0.32			–0.21	–0.60
Sex^*^Masc	1.05	3.10^∗∗^				
Sex^*^T	0.75	2.03^*^				
Sex^*^Masc^*^T	1.00	2.55^*^				
Progesterone (P)					0.52	1.57
Estradiol (E)					0.01	0.95
Masc^*^P					–0.24	–0.98
Masc^*^E					–0.40	−2.29^*^
T^*^P					–0.59	–1.94
Masc^*^T^*^P					–0.74	−2.52^*^
Sex^*^P					–1.08	–2.69^∗∗^

MRT, mental rotation task; VNT, virtual navigation task; RT, reaction time. ^∼^*p* < 0.10, ^*^p < 0.05, ^∗∗^*p* < 0.01, and ^∗∗∗^*p* < 0.001.

**TABLE 6:** Results of exploratory multiple regression models including interactions between biological sex, femininity, and sex hormones.

	**MRT accuracy**		**MRT RT**		**VNT RT**	
	**b**	**t**	**b**	**t**	**b**	**t**
Sex	–0.41	–4.02^∗∗∗^				
Femininity (Fem)			–0.01	–0.05	0.18	1.07
Testosterone (T)			0.20	1.17	–0.63	–3.37^∗∗^
Estradiol (E)			0.51	3.01^∗∗^	0.44	1.86^∼^
Fem^*^T			–0.29	–1.61	–0.55	−2.88^*^
Fem^*^E			0.07	0.40	0.35	1.87^∼^
T^*^E			0.22	1.65	0.21	1.58
Fem^*^T^*^E			0.67	3.07^∗∗^	0.58	2.27^*^
Progesterone (P)					0.15	0.57
Fem^*^P					–0.09	–0.44
T^*^P					–0.22	–0.94
Fem^*^T^*^P					0.77	3.33^∗∗^

MRT, mental rotation task; VNT, virtual navigation task; RT, reaction time. ^∼^*p* < 0.10, ^*^p < 0.05, ^∗∗^*p* < 0.01, and ^∗∗∗^*p* < 0.001.

**FIGURE 2:**
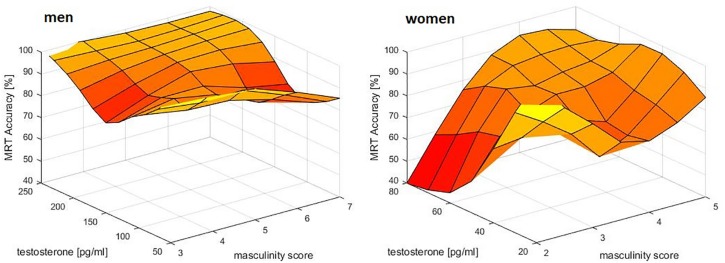
Relationship of masculinity and testosterone to mental rotation accuracy (MRT Accuracy). A combined effect of masculinity and testosterone was identified in women. Women with both, high masculinity and high testosterone levels had the highest MRT accuracy. In men a small, but non-significant positive association to testosterone was observed, but no effect of masculinity – probably due to a ceiling effect. Data were smoothed in the 3d space using the matlab function *gridfit*.

For mental rotation accuracy, a significant interaction between sex, masculinity and testosterone was observed (*b* = 1.00, SE_b_ = 0.39, *t* = 2.55, *p* = 0.01). Women with both high masculinity and high testosterone levels had the highest mental rotation accuracy, while in men only a trend toward higher accuracy with higher testosterone levels was visible (Figure 2).

For mental rotation reaction times (MRT RT), no significant interaction between masculinity and testosterone was observed, but masculinity and testosterone remained significant predictors in the model. While masculinity was negatively related to mental rotation reaction times (*b* = −0.28, SE_b_ = 0.13, *t* = −2.18, *p* = 0.03), testosterone was positively related to mental rotation reaction times (*b* = 0.39, SE_b_ = 0.13, *t* = 3.06, *p* = 0.003). Irrespective of their biological sex, participants with higher masculinity, but lower testosterone levels solved mental rotation items faster (Figure 1). Estradiol and progesterone did not survive as significant predictor in the model, highlighting testosterone as the hormone with the strongest effect on MRT RT.

**FIGURE 3:**
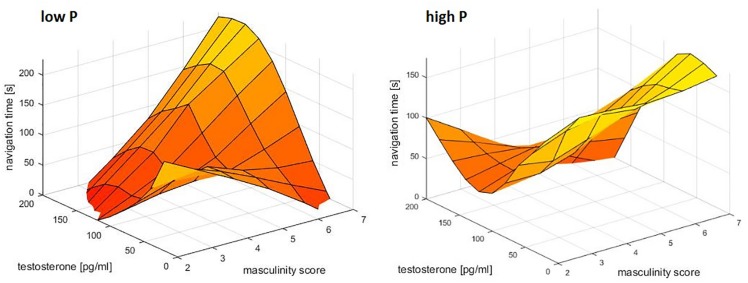
Relationship of masculinity and testosterone to navigation times. A combined effect of masculinity and testosterone was identified. In participants with high progesterone levels, high masculinity and high testosterone levels related to the fastest navigation times. In participants with low progesterone levels, the opposite pattern was observed. Data were smoothed in the 3d space using the matlab function *gridfit*.

For navigation times, the interaction between masculinity and testosterone was further qualified by progesterone (*b* = −0.74, SE_b_ = 0.29, *t* = −2.51, *p* = 0.01). This interaction resulted from the fact, that testosterone was most negatively related to navigation times in participants with high progesterone levels and high masculinity (Figure 3). In participants with low progesterone levels and high masculinity the opposite pattern was observed, i.e., a positive association between testosterone and navigation times. Accordingly in the absence of progesterone, testosterone improved navigation performance for participants with low masculinity, but impaired navigation performance for participants with high masculinity.

**FIGURE 4:**
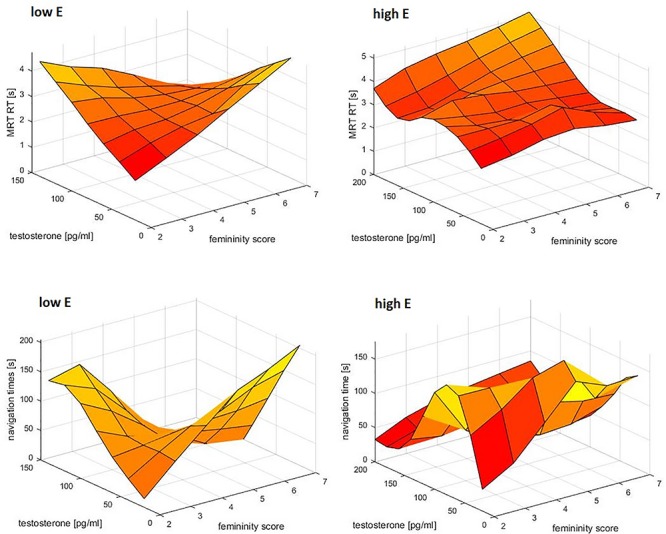
Interactive effect of estradiol, testosterone and femininity on response times in the mental rotation task (MRT) and navigation task. Testosterone related to response times for participants with low estradiol levels, depending on their femininity. Participants with low femininity showed a positive association between testosterone and response times, i.e., higher testosterone was related to slower reactions. Participants with high femininity showed a negative association between testosterone and response times, i.e., higher testosterone was related to faster reactions. Data were smoothed in the 3d space using the matlab function *gridfit*.

There was no interaction between femininity and sex hormones in the prediction of mental rotation accuracy. However, for both mental rotation reaction times and navigation times significant three-way interactions between femininity, testosterone and estradiol were observed (MRT: *b* = 0.68, SE_b_ = 0.22, *t* = 3.07, *p* = 0.003; VNT: *b* = 0.77, SE_b_ = 0.23, *t* = 3.33, *p* = 0.001). These 3-way interactions were accompanied by a two-way interaction between femininity^*^testosterone and a main effect of estradiol in the case of the MRT. For the MRT, but not for the VNT, estradiol was related to longer (slower) RT. The interactions are plotted in Figure 4. In both tasks associations between testosterone and reaction time were observed for participants with low estradiol levels, depending on their femininity. In participants with low femininity, testosterone showed a positive relationship to RT, i.e., the higher the testosterone levels, the slower the reactions. In participants with high femininity, testosterone showed a negative relationship to RT, i.e., the higher the testosterone levels, the faster the reactions. In addition, for navigation times the same interaction was observed for progesterone, i.e., interactive effects of femininity and testosterone on navigation times in participants with low progesterone levels.

## Discussion

The present study set out to investigate, whether gender role or sex hormones mediate sex differences in spatial performance. In addition we sought to explore potential interactive effects between gender role and sex hormones on spatial performance. We hypothesized highest spatial abilities in participants with both high masculinity and high testosterone levels. Furthermore we hypothesized stronger testosterone influences in participants with lower progesterone levels.

We found that neither gender role nor sex hormones or their ratios alone explained sex differences in spatial tasks. This was observed in both the mental rotation and the virtual navigation task, even though performance in both tasks was unrelated. This is in line with results of a previous study, demonstrating no effect of sex hormones on a similar navigation task (Harris et al., 2019). While sex differences in the mental rotation task emerged in accuracy, but not reaction times, sex differences in the navigation task emerged in response times. These differences may be due to the fact that a time limit was posed for the mental rotation task, but not for the navigation task, while all items had to be solved correctly in the navigation task, but not in the mental rotation task.

However, while none of the variables tested were able to explain the sex difference alone, sex did not remain a significant predictor in any of the multiple regression models assessing the interactive effects of gender role and sex hormones. This suggests that it’s their interactive effects that contribute to the differences observed between men and women, which is in line with psycho-biosocial models. Specifically, we observed the expected interaction between masculinity and testosterone for both performance measures that showed a sex difference, i.e., mental rotation accuracy and navigation times. While the interaction was qualified by biological sex for the MRT, it was qualified by progesterone in the VNT. In the MRT, a combined effect of masculinity and testosterone on accuracy was only observed in women. As expected, women with both, high masculinity scores and high testosterone levels showed the best performance. For navigation times the same effect was observed in participants with high progesterone levels, while the opposite effect was observed in participants with low progesterone levels. Since women have higher progesterone levels than men, both observations are in line with the assumption that progesterone modulates testosterone influences. They are, however, in the opposite direction as hypothesized. While we assumed that testosterone effects would be stronger in participants with lower progesterone levels due to progesterone’s higher affinity for the enzyme 5α-reductase, we found that testosterone and masculinity enhance spatial performance in participants with high progesterone levels (Figures 2, 3), but impair spatial performance in participants with low progesterone levels (Figure 3). This suggests different mechanisms of testosterone action in the presence or absence of progesterone. It can be assumed that in the absence of progesterone, testosterone mainly acts as dihydrotestosterone, while in the presence of high progesterone, testosterone does not get converted to dihydrotestosterone. In this case, testosterone can either act directly on androgen receptors or it acts as estradiol on estrogen receptors after conversion via the enzyme aromatase. This suggests different effects of dihydrotestosterone, testosterone and estradiol on spatial abilities and emphasizes dihydrotestosterone as an important sex hormone to consider in future studies. The fact that progesterone only emerged as a predictor for navigation times, as well as the fact that the combined effect of masculinity and testosterone on mental rotation accuracy was only observed in women, may be attributable to a ceiling effect in men (compare Figure 1). Almost all men reached an accuracy of over 90 percent, leaving little room for variation due to gender role or sex hormones.

Interestingly, associations of gender role and sex hormone to spatial performance also emerged for mental rotation reaction times – the one measure that did not show sex differences in spatial abilities. Irrespective of biological sex, masculinity, but not femininity, emerged as a predictor of mental rotation reaction times, such that more masculine individuals of either sex, showed faster reactions. This finding is in line with previous reports summarized in the meta-analysis by Reilly and Neumann (2013) and the effect size is in the range reported by Reilly and Neumann (2013). Note, however, that this effect only reached significance in the multiple regression model, when testosterone was also controlled for. Testosterone showed the opposite effect, i.e., individuals with higher testosterone levels were slower. While the correlation to testosterone was only significant in men, the effect survived across participants and did not interact significantly with biological sex. This is probably attributable to the overall lower testosterone levels in women. Likewise, in the correlation analyses, estradiol was related to slower reaction times across participants and progesterone was related to slower reaction times in men. Note, however, that the multiple regression analysis clearly identified testosterone as the hormone with the strongest influence, since neither estradiol nor progesterone survived as predictor in that model. The fact that masculinity and testosterone had opposite effects on mental rotation reaction times may explain, why no sex difference was found in this measure. Since masculinity includes personality traits like risk taking and competitiveness, its relationship to faster reactions seems plausible. The finding regarding testosterone levels, however, suggests, that testosterone may play a role in regulating the speed-accuracy trade-off participants are faced with during a timed task. This finding hints at the possibility of testosterone improving spatial performance by slowing reaction times, leading to more considerate decision making in the MRT. This idea is somewhat unexpected as testosterone has previously been shown to increase impulsive behavior (e.g., Agrawal et al., 2018) and better spatial performance (e.g., Hooven et al., 2004; Hausmann et al., 2009). However, u-shaped relationships and negative activational influences of testosterone on spatial performance have also been reported (e.g., Hromatko and Tadinac, 2006). On the contrary, estradiol has been discussed to decrease impulsive behavior (Howard et al., 1988, Diekhof, 2015, Roberts et al., 2018), which is in line with it’s relation to increased response times observed in the present study. Furthermore, the estradiol finding is in line with other studies suggesting a negative effect of estradiol on spatial performance in humans (e.g., Courvoisier et al., 2013; Hampson et al., 2014), but contrasts findings from animal studies, suggesting a positive effect of estradiol on spatial working memory (e.g., Williams et al., 1990; Healy et al., 1999, Workman et al., 2012). Note, however, that also in animal studies, negative findings and null effects regarding estradiol actions on spatial performance have been described and it has been discussed that estadiol actions may differ between different types of spatial abilities and depending on spatial strategy (Williams et al., 1990; Chesler and Juraska, 2000; Snihur et al., 2008; Lipatova and Toufexis, 2013).

However, the present study did not only identify masculinity to interact with sex hormone actions, but also femininity in a three-fold interaction of femininity, testosterone and estradiol on response times in both tasks (compare Figure 1). The fact that the association of testosterone to spatial response times is modulated by both estradiol and femininity may contribute to the mixed findings reported in the literature, where both positive and negative associations, as well as u-shaped relationships have been reported (e.g., Hooven et al., 2004; Driscoll et al., 2005; Halari et al., 2005; Falter et al., 2006; Hausmann et al., 2009; Puts et al., 2010; Courvoisier et al., 2013). Furthermore, this 3-fold interaction may help to shed light on the seemingly contradictory findings discussed in the previous paragraph. Including femininity in the model, revealed that testosterone related to response times in spatial tasks in participants with low estradiol levels, but depending on their femininity. These findings show that testosterone exerts its actions on response times (i) only in the absence of estradiol and (ii) in different directions for high and low femininity. In participants with low femininity, testosterone was related to slower reaction times, while in participants with higher femininity, testosterone was related to faster reaction times. Most importantly, this finding was consistent across the two spatial tasks employed in this study, even though response times revealed sex differences in the navigation task, but not in the mental rotation task.

Regarding (i), the fact that the interaction between femininity and testosterone only emerges for individuals with low estradiol levels may reflect the underlying biochemistry of these hormones. Testosterone is converted to estradiol via the enzyme aromatase. Accordingly across comparable testosterone levels, high estradiol may reflect higher aromatase activity, while low estradiol may reflect lower aromatase activity. Thus, in individuals with higher aromatase activity, estradiol may be the more relevant hormone to modulate performance, while in individuals with low aromatase activity, testosterone may be the more relevant hormone for modulating performance.

Regarding (ii), the interaction between femininity and testosterone highlights – for the first time – an important role for femininity in spatial abilities. The fact that this role is clearly modulatory may explain, why no associations between femininity and spatial performance were observed in previous studies (Reilly and Neumann, 2013). Linking this finding to the discussion in the previous paragraph regarding testosterones relationship to more impulsive decision making, it appears that personality traits associated with high femininity (e.g., expressivity, neuroticism) fuel this association, while low femininity reverses it. This finding adds to the discussion that sex hormones may have different effects on differently organized neural structures. However, it appears that gender role is a better proxy for how a brain is organized, since femininity survived as a predictor in the model, while biological sex did not.

While of course most participants with high femininity were female and most participants with low femininity were male, we specifically identified 14 participants, whose gender role did not correspond to their biological sex. Using a median cut-off, three men showed high masculinity but low femininity, i.e., a typically female gender role, while four women showed low masculinity but high femininity, i.e., a typically male gender role. Furthermore, several participants showed an indifferent (low masculinity and low femininity, 2 men) or androgynous (high masculinity and high femininity, 2 men, 3 women) gender role pattern.

In summary, results of the present study suggest, that neither gender role nor sex hormones alone mediate sex differences in spatial performance. Rather it seems that their contributions to spatial performance are mostly combinatory and interactive. While masculinity seems to boost testosterone effects in those tasks that show significant sex differences, femininity modulates testosterone effects on response times. The combined effect of masculinity and testosterone was modulated by progesterone, while the interactive effect of femininity and testosterone was modulated by estradiol levels.

## Data Availability

All datasets generated for this study are included in the manuscript and/or the supplementary files.

## Ethics Statement

The study was approved by the University of Salzburg’s ethics committee and conforms to the Code of Ethics of the World Medical Association (Declaration of Helsinki). Informed written consent was obtained from all participants.

## Author Contributions

BP designed the study, analyzed the data, and wrote the manuscript. JS and LvL collected the data and assisted in the literature research. TH developed the navigation task, provided the VR equipment, and assisted in the data collection.

## Conflict of Interest Statement

The authors declare that the research was conducted in the absence of any commercial or financial relationships that could be construed as a potential conflict of interest.

## References

[B1] Agrawal J. Ludwig B. Roy B. Dwivedi Y. (2018). Chronic testosterone increases impulsivity and influences the transcriptional activity of the alpha-2a adrenergic receptor signaling pathway in rat brain. *Mol. Neurobiol.* 56 4061–4071. 10.1007/s12035-018-1350-z 30264294 PMC6502699

[B2] Andreano J. M. Cahill L. (2009). Sex influences on the neurobiology of learning and memory. *Learn. Mem.* 16 248–266. 10.1101/lm.918309 19318467

[B3] Barth C. Villringer A. Sacher J. (2015). Sex hormones affect neurotransmitters and shape the adult female brain during hormonal transition periods. *Front. Neurosci.* 9:37 10.3389/fnins.2015.00037 PMC4335177 25750611

[B4] Bem S. L. (1974). The measurement of psychological androgyny. *J. Consul. Clin. Psychol.* 42 155–162. 4823550

[B5] Blum R. W. MMari K. Moreau C. (2017). It begins at 10. How gender expectations shape early adolescence around the word. *J. Adoles. Health* 61 S3–S4. 10.1016/j.jadohealth.2017.07.009 PMC5612023 28915989

[B6] Chesler E. J. Juraska J. M. (2000). Acute administration of estrogen and progesterone impairs the acquisition of the spatial Morris water maze in ovariectomized rats. *Horm. Behav.* 38 234–242. 10.1006/hbeh.2000.1626 11104641

[B7] Choi N. Fuqua D. R. (2003). The structure of the bem sex role inventory: a summary report of 23 validation studies. *Educ. Psychol. Measure.* 63 872–887. 10.1177/0013164403258235

[B8] Courvoisier D. S. Renaud O. Geiser C. Paschke K. Gaudy K. Jordan K. (2013). Sex hormones and mental rotation: An intensive longitudinal investigation. *Horm. Behav.* 63 345–351. 10.1016/j.yhbeh.2012.12.007 23261859

[B9] Diekhof E. K. (2015). Be quick about it. Endogenous estradiol level, menstrual cycle phase and trait impulsiveness predict impulsive choice in the context of reward acquisition. *Horm. Behav.* 74 186–198. 26092059 10.1016/j.yhbeh.2015.06.001

[B10] Driscoll I. Hamilton D. A. Yeo R. A. Brooks W. M. Sutherland R. J. (2005). Virtual navigation in humans: the impact of age, sex, and hormones on place learning. *Horm. Behav.* 47 326–335. 10.1016/j.yhbeh.2004.11.013 15708762

[B11] Eagly A. H. Koenig A. M. (2006). *Social Role Theory of Sex Differences and Similarities Implication for Prosocial Behavior.* New Jersey, NJ: Lawrence Erlbaum Associates Publishers.

[B12] Falter C. M. Arroyo M. Davis G. J. (2006). Testosterone: activation or organization of spatial cognition? *Biol. Psychol.* 73 132–140. 10.1016/j.biopsycho.2006.01.011 16490297

[B13] Fehring R. J. Schneider M. Raviele K. (2006). Variability in the phases of the menstrual cycle. *J. Obstet. Gynecol. Neonat. Nurs.* 35 376–384. 10.1111/j.1552-6909.2006.00051.x 16700687

[B14] Frick A. Ferrara K. Newcombe N. S. (2013a). Using a touch screen paradigm to assess the development of mental rotation between 3$1/2$ and 5$1/2$ years of age. *Cogn. Process* 14 117–127. 10.1002/wcs.1380 23306783

[B15] Frick A. Hansen M. A. Newcombe N. S. (2013b). Development of mental rotation in 3-to 5-year-old children. *Cogn. Dev.* 28 386–399. 10.1016/j.cogdev.2013.06.002

[B16] Ganis G. Kievit R. (2015). A new set of three-dimensional shapes for investigating mental rotation processes: validation data and stimulus set. *J. Open Psychol. Data* 3:e3 10.5334/jopd.ai

[B17] Gingnell M. Comasco E. Oreland L. Fredrikson M. Sundström-Poromaa I. (2010). Neuroticism-related personality traits are related to symptom severity in patients with premenstrual dysphoric disorder and to the serotonin transporter gene-linked polymorphism 5-HTTPLPR. *Arch. Womens Ment. Health* 13 417–423. 10.1007/s00737-010-0164-4 20440524 PMC2941046

[B18] Gruber F. Scherndl T. Ortner T. Pletzer B. (2019). *Psychometric Properties of the Multifaceted Gender-Related Attributes Survey (GERAS) European Journal of Psychological Assessment.* Brussel: European Association of Psychological Assessment. 10.1027/1015-5759/a000528 PMC7116055 32913384

[B19] Halari R. Hines M. Kumari V. Mehrotra R. Wheeler M. Ng V. (2005). Sex differences and individual differences in cognitive performance and their relationship to endogenous gonadal hormones and gonadotropins. *Behav. Neurosci.* 119 104–117. 10.1037/0735-7044.119.1.104 15727517

[B20] Halbreich U. Lumley L. A. Palter S. Manning C. Gengo F. Joe S. H. (1995). Possible acceleration of age effects on cognition following menopause. *J. Psychiatr. Res.* 29:153. 10.1016/0022-3956(95)00005-p 7473292

[B21] Halpern D. F. (2000). *Sex Differences in Cognitive Abilities.* Hoboken: John Wiley & Sons, Ltd.

[B22] Hampson E. (1990). Variations in sex-related cognitive abilities across the menstrual cycle. *Brain Cogn.* 14 26–43. 10.1016/0278-2626(90)90058-v 2223043

[B23] Hampson E. Levy-Cooperman N. Korman J. M. (2014). Estradiol and mental rotation: relation to dimensionality, difficulty, or angular disparity? *Horm. Behav.* 65 238–248. 10.1016/j.yhbeh.2013.12.016 24394702

[B24] Harris T. Scheuringer A. Pletzer B. (2019). Perspective and strategy interactively modulate sex differences in navigation. *Biol. Sex Differ*. 10:17. 10.1186/s13293-019-0232-z PMC6451294 30954081

[B25] Hausmann M. Schoofs D. Rosenthal H. E. Jordan K. (2009). Interactive effects of sex hormones and gender stereotypes on cognitive sex differences - a psychobiosocial approach. *Psychoneuroendocrinology* 34 389–401. 10.1016/j.psyneuen.2008.09.019 18992993

[B26] Hausmann M. Slabbekoorn D. Van Goozen S. H. Cohen-Kettenis P. T. Güntürkün O. (2000). Sex hormones affect spatial abilities during the menstrual cycle. *Behav. Neurosci.* 114 1245–1250. 10.1037//0735-7044.114.6.1245 11142657

[B27] Healy S. D. Braham S. R. Braithwaite V. A. (1999). Spatial working memory in rats: no differences between the sexes. *Proc. R. Soc. London B Biol. Sci.* 266 2303–2308. 10.1098/rspb.1999.0923 10629980 PMC1690445

[B28] Hooven C. K. Chabris C. F. Ellison P. T. Kosslyn S. M. (2004). The relationship of male testosterone to components of mental rotation. *Neuropsychologia* 42 782–790. 10.1016/j.neuropsychologia.2003.11.012 15037056

[B29] Howard R. Gifford M. Lumsden J. (1988). Changes in an electrocortical measure of impulsivity during the menstrual cycle. *Pers. Individ. Diff.* 9 5 917–918. 10.1016/0191-8869(88)90010-4

[B30] Hromatko I. Tadinac M. (2006). Testosterone levels influence spatial ability: Further evidence for curvilinear relationship. *Rev. Psychol.* 13 27–34.

[B31] Kelly S. J. Ostrowski N. L. Wilson M. A. (1999). Gender differences in brain and behavior: hormonal and neural bases. *Pharmacol. Biochem. Behav.* 64 655–664. 10.1016/S0091-3057(99)00167-7 10593187

[B32] Lauer J. E. Udelson H. B. Jeon S. O. Lourenco S. F. (2015). An early sex difference in the relation between mental rotation and object preference. *Front. Psychol.* 6:558. 10.3389/fpsyg.2015.00558 26005426 PMC4424807

[B33] Leplow B. Lehnung M. Pohl J. Herzog A. Ferstl R. Mehdorn M. (2003). Navigational place learning in children and young adults as assessed with a standardized locomotor search task. *Br. J. Psychol.* 94 299–317. 10.1348/000712603767876244 14511545

[B34] Levine S. C. Foley A. Lourenco S. Ehrlich S. Ratliff K. (2016). Sex differences in spatial cognition: advancing the conversation. *Wiley Interdiscip. Rev. Cogn. Sci.* 7 127–155. 10.1002/wcs.1380 26825049

[B35] Levine S. C. Huttenlocher J. Taylor A. Langrock A. (1999). Early sex differences in spatial skill. *Dev. Psychol.* 35:940. 10.1037//0012-1649.35.4.940 10442863

[B36] Linn M. C. Petersen A. C. (1985). Emergence and characterization of sex differences in spatial ability: a meta-analysis. *Child Dev.* 56 1479–1498. 10.2307/1130467 4075870

[B37] Lipatova O. Toufexis D. J. (2013). Estrogen enhances the retention of spatial reference memory in the open field tower task, but disrupts the expression of spatial memory following a novel start position. *Neurobiol. Learn. Mem.* 99 50–58. 10.1016/j.nlm.2012.11.002 23178325

[B38] Moore D. S. Johnson S. P. (2008). Mental rotation in human infants a sex difference. *Psychol. Sci.* 19 1063–1066. 10.1111/j.1467-9280.2008.02200.x 19076473 PMC2651884

[B39] Moore D. S. Johnson S. P. (2011). Mental rotation of dynamic, three-dimensional stimuli by 3-month-old infants. *Infancy* 16 435–445. 10.1111/j.1532-7078.2010.00058.x 26312057 PMC4547474

[B40] Nash S. C. (1979). “Sex role as mediator of intellectual functioning,” in *Sex-Related Differences in Cognitive Functioning Developmental Issues*, eds Wittig M. A. Petersen A. C. (New York, NY: Academic Press), 263–302.

[B41] Pletzer B. (2019). Sex hormones and gender role relate to grey matter volumes in sexually dimorphic brain areas. *Front. Neurosci.* 13:592 10.3389/fnins.2019.00592 PMC6591487 31275099

[B42] Pletzer B. Petasis O. Ortner T. Cahill L. (2015). Interactive effects of culture and sex hormones on the sex role self-concept. *Front. Neurosci.* 9:240. 10.3389/fnins.2015.00240 26236181 PMC4500910

[B43] Puts D. A. Cárdenas R. A. Bailey D. H. Burriss R. P. Jordan C. L. Breedlove S. M. (2010). Salivary testosterone does not predict mental rotation performance in men or women. *Horm. Behav.* 58 282–289. 10.1016/j.yhbeh.2010.03.005 20226788

[B44] Quinn P. C. Liben L. S. (2008). A sex difference in mental rotation in young infants. *Psychol. Sci.* 19 1067–1070. 10.1111/j.1467-9280.2008.02201.x 19076474

[B45] Raven J. C. Raven J. C. Court J. H. (1962). *Advanced Progressive Matrices.* London: HK Lewis.

[B46] Reilly D. Neumann D. L. (2013). Gender-role differences in spatial ability: a meta-analytic review. *Sex Roles* 68 521–535. 10.1007/s11199-013-0269-0

[B47] Roberts B. Eisenlohr-Moul T. Martel M. M. (2018). Reproductive steroids and ADHD symptoms across the menstrual cycle. *Psychoneuroendocrinology* 88 105–114. 10.1016/j.psyneuen.2017.11.015 29197795 PMC5803442

[B48] Silverman I. Eals M. (1992). *Sex Differences in Spatial Abilities: Evolutionary Theory, and Data.* New York, NY: Oxford University Press.

[B49] Sitruk-Ware R. (2006). New progestagens for contraceptive use. *Hum. Reprod. Update* 12 169–178. 10.1093/humupd/dmi046 16291771

[B50] Snihur A. W. K. Hampson E. Cain D. P. (2008). Estradiol and corticosterone independently impair spatial navigation in the Morris water maze in adult female rats. *Behav. Brain Res.* 187 56–66. 10.1016/j.bbr.2007.08.023 17913254

[B51] Stenbæk D. S. Budtz-Jørgensen E. Pinborg A. Jensen P. S. Frokjaer V. G. (2019). Neuroticism modulates mood responses to pharmacological sex hormone manipulation in healthy women. *Psychoneuroendocrinology* 99 251–256. 10.1016/j.psyneuen.2018.10.016 30390443

[B52] Tingley D. Yamamoto T. Hirose K. Keele L. Imai K. Yamamoto M. T. (2017). *Package ‘Mediation’.* https://imai.fas.harvard.edu/projects/mechanisms.html. (accessed March 17, 2019).

[B53] Titze C. Jansen P. Heil M. (2010). Mental rotation performance and the effect of gender in fourth graders and adults. *Eur. J. Dev. Psychol.* 7 432–444. 10.1080/17405620802548214

[B54] Voyer D. Postma A. Brake B. Imperato-McGinley J. (2007). Gender differences in object location memory: a meta-analysis. *Psycho. Bull. Rev.* 14 23–38. 10.3758/bf03194024 17546728

[B55] Voyer D. Voyer S. Bryden M. P. (1995). Magnitude of sex differences in spatial abilities: a meta-analysis and consideration of critical variables. *Psychol. Bull.* 117:250. 10.1037//0033-2909.117.2.250 7724690

[B56] Williams C. L. Barnett A. M. Meck W. H. (1990). Organizational effects of early gonadal secretions on sexual differentiation in spatial memory. *Behav. Neurosci.* 104 84–97. 10.1037/0735-7044.104.1.84 2317288

[B57] Workman J. L. Barha C. K. Galea L. A. (2012). Endocrine substrates of cognitive and affective changes during pregnancy and postpartum. *Behav. Neurosci.* 126:54. 10.1037/a0025538 21967374

